# Alzheimer’s Disease: Recent Developments in Pathogenesis, Diagnosis, and Therapy

**DOI:** 10.3390/life15040549

**Published:** 2025-03-27

**Authors:** Ivana Shawkatova, Juraj Javor

**Affiliations:** Institute of Immunology, Faculty of Medicine, Comenius University in Bratislava, Odborarske namestie 14, 811 08 Bratislava, Slovakia; juraj.javor@fmed.uniba.sk

As the leading cause of dementia, Alzheimer’s disease (AD) remains one of the most pressing global health challenges, affecting millions worldwide and placing an immense burden on healthcare systems and caregivers. Despite significant advancements in understanding its pathogenesis, diagnosis, and potential therapeutic strategies, AD continues to be an incurable neurodegenerative disorder with complex multifactorial etiology [1]. This Special Issue of *Life* aimed to collect original research and review papers that provide a comprehensive update on recent developments in AD research and highlight new insights into its underlying mechanisms, innovative diagnostic techniques, and emerging therapeutic strategies. We believe that the included contributions offer novel perspectives on the intricate relationship between systemic health and neurodegeneration, the role of metabolic and sex-related factors in hippocampal function, advancements in imaging techniques, and potential pharmacological interventions.

Alzheimer’s disease affects predominantly elderly people, causing a progressive deterioration in memory and cognitive skills. Based on the age at onset and type of heritability, two forms of AD exist: early-onset and late-onset Alzheimer’s disease (LOAD). The vast majority of AD cases are represented by the sporadic LOAD form, in which symptoms become apparent at an age of around the mid-60s and later. While the apolipoprotein E gene (*APOE*) ε4 allele and aging are considered the most important risk factors of AD, additional genetic, intrinsic, environmental, and lifestyle factors also contribute to the disease development [2,3,4,5]. Women have significantly higher AD prevalence and exhibit faster cognitive decline compared to men, suggesting that sex-based anatomic, hormonal, and metabolic differences are crucial factors influencing AD development and progression [6,7]. In their review (Contribution 1), Martínez-Martos et al. discuss the critical role of the hippocampus in cognition and analyze how sex differences influence its structure and function. The authors highlight the significance of glucose metabolism and insulin regulation, particularly through glucose transporters GLUT3 and GLUT4. Neurogenesis, dendritic spine density, and electrophysiological plasticity in the hippocampus are shown to be influenced by sex hormones, with estrogen playing a neuroprotective role. The authors emphasize that postmenopausal women, who experience a sharp decline in estrogen levels, exhibit increased vulnerability to hippocampal atrophy and metabolic dysregulation. Furthermore, genetic variants affecting estrogen synthesis, such as polymorphisms in the aromatase enzyme, may contribute to sex-related differences in AD susceptibility. Another key point discussed in this review is the interplay between insulin resistance and AD risk. The authors explore how the insulin-sensitive glucose transporter GLUT4 and the insulin-regulated aminopeptidase IRAP are involved in hippocampal energy metabolism. Since the dysregulation of these systems has been linked to type 2 diabetes, and given the growing recognition of AD as “type 3 diabetes”, understanding the sex differences in hippocampal metabolism is crucial. Future research focusing on personalized metabolic interventions may improve therapeutic outcomes for AD patients.

Another intriguing topic in AD research involves the potential link between systemic infections and neurodegeneration. In their review (Contribution 2), Shawkatova et al. delve into the growing evidence connecting periodontal disease to AD pathogenesis. The review provides an in-depth examination of *Porphyromonas gingivalis*, a key periodontal pathogen, and its involvement in neuroinflammatory processes, amyloid-β accumulation, and tau hyperphosphorylation. The role of virulence factors, such as gingipains and lipopolysaccharides, in the disruption of the blood–brain barrier and neurotoxicity is thoroughly explored. The authors also discuss emerging research suggesting that *P. gingivalis*-derived outer membrane vesicles (OMVs) serve as potential mechanisms for transporting neurotoxic components into brain tissues. Additionally, systemic inflammation resulting from chronic periodontal infections is hypothesized to exacerbate neurodegenerative processes through cytokine release and microglial activation. Given that some studies have detected *P. gingivalis* virulence factors in the brains of AD patients, further research is needed to elucidate whether managing periodontal disease can effectively reduce AD risk. This review underscores the importance of interdisciplinary research between oral health and neurology and highlights the potential for preventive strategies targeting periodontal disease.

The diagnosis of AD relies on a combination of clinical assessment, cognitive testing, and biomarker analysis, including cerebrospinal fluid markers and advanced neuroimaging techniques. Neuroimaging, particularly amyloid positron emission tomography (PET), tau PET, and MRI, plays a crucial role in detecting pathological brain changes, enabling early diagnosis and helping to monitor disease progression and differentiate AD from other dementias [8,9]. However, amyloid PET imaging, despite being a powerful tool, suffers from resolution limitations that impact its precision. In their research article (Contribution 3), Shah et al. present a novel artificial intelligence-driven approach to improving PET scan resolution. Their latent diffusion model for resolution recovery (LDM-RR) enhances PET quantification accuracy, reduces inter-tracer variability, and increases sensitivity to subtle amyloid-β changes over time. The authors describe their methodology, leveraging a synthetic data generation pipeline to create high-resolution PET digital phantoms for model training. By integrating MRI-guided super-resolution techniques, the model improves amyloid PET quantification by addressing partial volume effects. The study demonstrates that deep-learning methodologies have the potential to revolutionize neuroimaging and improve early detection capabilities in AD research, paving the way for more accurate and reliable biomarker assessments. Given the growing reliance on imaging biomarkers for clinical trials and therapeutic interventions, these advancements hold promise for refining patient stratification and treatment monitoring. Further research is warranted to integrate such machine learning approaches into routine clinical workflows.

Symptomatic treatments such as cholinesterase inhibitors and memantine remain integral to managing cognitive and functional symptoms in AD patients. Recently, AD therapy has advanced with the approval of monoclonal antibodies like lecanemab and donanemab, which target amyloid-β plaques to slow cognitive decline in early-stage patients [10,11,12]. In the search for effective AD treatments, drug repurposing has also gained traction as a promising strategy. Recently, an anticancer drug called nilotinib, a tyrosine kinase inhibitor initially developed for chronic myeloid leukemia, has shown potential neuroprotective effects by enhancing autophagy in the CNS through targeting the c-Abl signaling pathway, ultimately lowering both amyloid plaque and hyperphosphorylated tau protein burden in AD brains and attenuating the loss of hippocampal brain volume [12,13]. In their research article (Contribution 4), Srivastava et al. further explore this concept by investigating the effects of nilotinib on amyloid-β processing and mitochondrial function in SH-SY5Y neuroblastoma cells. Interestingly, treatment with nilotinib did not change the expression of the key genes involved in amyloid-β formation, neuronal health, or mitochondrial functioning, while transmission electron microscopy (TEM) images revealed the potential detrimental effects of nilotinib on mitochondrial morphology in SH-SY5Y cells. Although these results do not strongly support the efficacy of nilotinib as a neuroprotective agent, they underscore the need for further research to provide insights into the complexity of drug repurposing efforts in AD treatment.

Predicting AD and other dementias remains a critical challenge in clinical practice and public health. Advancements in machine learning, artificial intelligence, and biomarker analysis hold promise to significantly enhance the ability to predict AD in its early stages. These innovations offer the potential for early interventions and improved management strategies for individuals at risk of developing AD [14,15]. In their review article (Contribution 5), Tang et al. provide a comprehensive synthesis of existing dementia risk prediction models, focusing on their applicability to different disease-specific groups. The authors emphasize that while several models have been developed, most fail to incorporate disease-specific risk factors. The review discusses validated models for populations with diabetes and cardiovascular diseases and underlines the need for broader application across other at-risk groups. A key takeaway from this review is the necessity of tailoring risk prediction to account for unique comorbidities and lifestyle factors. This approach could enhance early intervention strategies and facilitate personalized risk assessments in clinical settings.

The articles featured in this Special Issue collectively advance our understanding of AD pathogenesis, diagnosis, and treatment strategies. The integration of systemic health factors, sex-based metabolic differences, and novel imaging techniques underscores the interdisciplinary nature of AD research. Additionally, the exploration of pharmacological interventions and predictive risk models highlights the complexity of AD and the need for a multifaceted approach to tackling this disease. We hope these contributions inspire further investigation and collaboration in the ongoing effort to unravel the complexities of AD and find effective interventions to combat this devastating disease.
